# Comparison of Laparoscopy and Laparotomy in the Treatment of Early Stage Endometrioid Endometrial Adenocarcinoma

**DOI:** 10.7759/cureus.79575

**Published:** 2025-02-24

**Authors:** Ilknur Cinar Dura, Mustafa Cengiz Dura, Miğraci Tosun, Handan Çelik, Hatice Nilden Arslan, Arif Kökçü

**Affiliations:** 1 Obstetrics and Gynaecology, Ondokuz Mayıs University Faculty of Medicine, Samsun, TUR; 2 Obstetrics and Gynaecology, Bakırköy Sadi Konuk Education and Research Hospital, Istanbul, TUR; 3 Public Health, Ondokuz Mayıs University Faculty of Medicine, Samsun, TUR; 4 Obstetrics and Gynaecology, ​​​​​​​VM Medical Park, Samsun, TUR

**Keywords:** abdominal hysterectomy, early-stage endometrial cancer, laparatomy, laparoscopic hysterectomy, survival

## Abstract

Objective

This study aims to compare the surgical and postoperative outcomes, as well as the safety and efficacy, of laparoscopic surgery and laparotomy in patients diagnosed with early-stage endometrioid endometrial cancer, focusing on operative time, intraoperative blood transfusion requirements, postoperative complications, and recurrence rates.

Materials and methods

Data were collected on patients who underwent surgery at the Department of Obstetrics and Gynaecology, Ondokuz Mayıs University Faculty of Medicine, with a diagnosis of endometrial cancer between January 2007 and December 2018. Patients were assessed using the staging approach proposed by FIGO in 2009. Patients with endometrioid adenocarcinoma histology in stages IA, IB, and II were chosen; 69 underwent laparoscopic surgery, and 133 underwent laparotomy. Patient survival rates, cancer recurrence, demographic information, and intraoperative and postoperative outcomes were compared.

Results

There was no significant difference between the groups in terms of survival and disease-free survival. The tumor size was larger in the laparotomy group, which may be attributed to preoperative imaging and clinical assessment guiding the surgical approach. The laparoscopy group had a shorter postoperative hospital stay, required less intraoperative blood transfusion, and experienced fewer postoperative complications. However, the operation time was shorter in the laparotomy group, likely due to the technical complexity of laparoscopy, which typically requires longer preparation and instrument handling times. While laparoscopy provides magnification that enhances visualization and access to lymph nodes, the number of pelvic and total lymph nodes removed was higher in the laparotomy group, possibly reflecting the standard surgical approach used in open procedures. These outcomes were compared and evaluated between the two surgical methods. All surgeries were performed by surgeons with similar levels of expertise in both techniques.

Discussion

Although the duration of the laparoscopy method is long, it has advantages such as short hospital stay, less intraoperative blood requirement, fewer post-operative complications, and early return to daily activities. There is no difference between both methods in terms of cancer recurrence and survival. The mean, 5-year survival and disease-free survival of the patients were similar.

Conclusion

Laparoscopic surgery is as safe and effective as laparotomy in the treatment of early-stage endometrial cancer. While this study reinforces an already well-established fact, it is important to acknowledge the continuous advancements in minimally invasive surgical techniques, including robotic surgery and vaginal natural orifice transluminal endoscopic surgery (vNOTES). Future research should focus on comparing these emerging techniques to further refine surgical approaches and optimize patient outcomes.

## Introduction

Endometrial cancer is the most common gynaecological malignancy in developed countries and the second most common gynaecological malignancy after cervical cancer in developing countries [1].

Endometrial cancer, also known as cancer of the lining of the uterus, is the sixth most common disease globally in terms of diagnoses and ranks 14th in terms of cancer-related fatalities in women, accounting for 320,000 new cases and 76,000 deaths in 2012 [2]. In low- and middle-income nations, a woman's chance of endometrial cancer by the age of 65 is 0.46%, but in high-income countries, it is 0.92% [3]. This disparity may be attributed to differences in obesity prevalence, hormone replacement therapy (HRT) usage, and screening practices, which are more common in high-income countries and are known risk factors for endometrial cancer.

The current standard therapy for women with endometrial cancer is a hysterectomy, which includes removing the uterus as well as the fallopian tubes and ovaries. Adjuvant radiation and chemotherapy are two more treatment choices. Pelvic/para-aortic lymph node (LN) dissection, with or without omental biopsy, is suggested for high-grade malignancies, tumors with poor histological characteristics, and tumors that infiltrate the myometrium [4]. Endometrial cancer surgery is typically performed via laparotomy [5].

While laparotomy is widely used in endometrial cancer surgery at many medical facilities, laparoscopic surgery is preferred in some cases, especially for early-stage cancers [6]. Many studies concur that the laparoscopic technique is superior to open surgery in terms of shorter hospital stays, fewer perioperative problems, and less intraoperative blood loss in short-term outcomes; however, long-term outcomes, including overall survival, disease-free survival, and cancer recurrence rates, have been found to be comparable between the two techniques [7].

The purpose of this study was to look into the postoperative outcomes of the surgical methods - laparoscopy and laparotomy - used in the treatment of patients diagnosed with early-stage endometrioid endometrial adenocarcinoma between January 2007 and December 2018 in the Department of Obstetrics and Gynaecology at Ondokuz Mayıs University Faculty of Medicine. Despite significant advancements in minimally invasive surgical techniques, the optimal approach for early-stage endometrial cancer remains a topic of debate. This study aims to address this clinical gap by directly comparing laparoscopic and laparotomic techniques in terms of oncological safety and perioperative outcomes, contributing to the ongoing discussion on the most effective surgical strategy in routine practice.

In addition, we intended to compare overall survival and disease-free survival over a 5-year period, as well as investigate the role and effectiveness of laparoscopy in the treatment of early-stage endometrial cancer.

## Materials and methods

This retrospective cohort study was conducted at Ondokuz Mayıs University Department of Obstetrics and Gynaecology from January 2007 to December 2018. The study included patients diagnosed with early-stage endometrioid endometrial adenocarcinoma who underwent laparoscopic or laparotomic surgery. The study protocol was approved by the Ondokuz Mayıs University Clinical Research Ethics Committee (Approval number: 2021/60, January 29, 2021).

The medical records of 202 patients with early-stage endometrioid adenocarcinoma (Stage IA, IB, and II) were reviewed. Patients diagnosed with non-endometrioid adenocarcinoma or Stage III/IV endometrial cancer were excluded from the study. Patients were assigned to either the laparotomy or laparoscopy group based on preoperative clinical evaluations, surgeon recommendations, and patient preferences, taking into account factors such as BMI, comorbidities, and tumor characteristics.

All procedures were performed by three senior gynecologic oncologists with equivalent expertise in both laparoscopic and open surgery techniques. The surgical team worked collaboratively, ensuring standardized procedures across both intervention groups. The laparoscopic procedure involved standard trocar placement, pneumoperitoneum establishment, and total laparoscopic hysterectomy with bilateral salpingo-oophorectomy (BSO), with or without lymphadenectomy, depending on oncological indications. The laparotomic approach consisted of a midline laparotomy with similar surgical steps performed via an open technique.

This study aimed to compare the laparoscopic and laparotomic approaches based on multiple parameters, including demographic characteristics (age, menopausal status, BMI, gravidity, and parity), co-existing conditions (menopause and diabetes), tumor characteristics (histological grades, myometrial invasion, lymphovascular invasion, and stage of lesion), intraoperative factors (operation duration, intraoperative complications, and blood transfusion requirements), postoperative factors (hospital stay, postoperative complications, and recurrence rates), and survival outcomes (5-year overall survival and disease-free survival).

The follow-up period for survival analysis began on the operation date, with the final follow-up recorded on March 30, 2021. Disease-free survival (DFS) was defined as the time between surgery and the first recurrence.

A total of 69 patients underwent laparoscopic surgery, while 133 underwent laparotomy. Among the laparoscopy group, 13 patients underwent laparoscopic hysterectomy (LH) and BSO, 22 underwent LH+BSO+pelvic lymph node dissection (LND), and 34 underwent LH+BSO+paraaortic+pelvic LND (PPLND). In the laparotomy group, 36 patients underwent total abdominal hysterectomy (TAH)+BSO, 21 underwent TAH+BSO+pelvic LND, and 76 underwent TAH+BSO+PPLND. Two patients initially assigned to laparoscopy required conversion to laparotomy due to postoperative hemorrhage and ureteral damage. These cases were analyzed within the laparoscopic group based on an intention-to-treat approach.

While the study follows a standardized surgical and analytical approach, these methods may be adapted based on institutional resources, surgeon expertise, and patient selection criteria. In centers equipped with robotic surgical systems, minimally invasive approaches may further improve operative efficiency. Additionally, patient-specific factors, such as BMI and comorbidities, can influence surgical modality selection and perioperative management strategies. These considerations should be taken into account when applying our methodology to different clinical settings.

The data were analyzed using IBM SPSS v23 (IBM Corp., Armonk, NY, USA). The Kolmogorov-Smirnov and Shapiro-Wilk tests were employed to assess the normality of the data distribution. Chi-square and Fisher’s Exact tests were applied to compare categorical variables between the two surgical groups, while the Mann-Whitney U test was used for non-normally distributed continuous variables. The Log Rank (Mantel-Cox) test was performed to evaluate overall survival and disease-free survival. For continuous variables, the results were expressed as mean ± standard deviation (SD), while categorical variables were presented as median (min-max). A p-value of <0.05 was considered statistically significant.

## Results

Group 1 consisted of 69 patients who had a laparoscopy, while Group 2 consisted of 133 patients who had a laparotomy. Participants' ages ranged from 35 to 85 years. The mean age was 55.9 ± 9.4 years for the laparoscopy group and 57.1 ± 10.3 years for the laparotomy group, with no statistically significant difference (p=0.418) (Table 1).

**Table 1:** Demographic characteristics of the groups. ^1^Independent samples t-test, ^2^Mann-Whitney U test

	Group 1 (n: 69)		Group 2 (n: 133)		p
	Mean ± SD	Median (min. - max.)	Mean ± SD	Median (min. - max.)	
Age	55.9 ± 9.4	56.0 (35.0 - 80.0)	57.1 ± 10.3	57.0 (37.0 - 85.0)	0.418^1^
Gravida	3.5 ± 2.0	4.0 (0 - 8.0)	3.3 ± 2.1	3.0 (0 - 10.0)	0.298^2^
Parity	2.8 ± 1.6	3.0 (0 - 7.0)	2.9 ± 1.9	3.0 (0 - 9.0)	0.828^2^

Among the participants, 146 were postmenopausal, with 52 in the laparoscopy group and 94 in the laparotomy group, showing no significant difference (p=0.480) (Table 2). Gravidity ranged from 0 to 10, and parity ranged from 0 to 9. The median gravidity was 4 for the laparoscopy group and 3 for the laparotomy group. The parity medians were both 3, with no statistically significant differences between the groups (gravidity: p=0.298; parity: p=0.828). There was no statistically significant difference in BMI between the laparoscopy and laparotomy groups (p=0.418).

**Table 2:** Menopausal and diabetes mellitus characteristics of the cases. ^1^Chi-square test

	Group 1 (n: 69)	Group 2 (n: 133)	Total	p^1^
Menopausal Status				
Yes	52 (75.4)	94 (70.7)	146 (72.3)	0.480
No	17 (24.6)	39 (29.3)	56 (27.7)	
Diabetes Mellitus				
No	49 (71)	85 (63.9)	134 (66.3)	0.311
Yes	20 (29)	48 (36.1)	68 (33.7)	

In Group 1, the surgery took 60 to 495 minutes on average, with a median of 240 minutes. Group 2's duration had a median of 135 minutes and varied from 45 to 320 minutes. The group that underwent laparoscopy had an operation that lasted longer, with a statistically significant difference (p<0.001). The length of the postoperative hospital stay varied from 1 to 22 days, with Group 2's hospital stay being statistically considerably longer than Group 1's following surgery. The median in Group 1 was 4.0 (range: 1-12), whereas the median in Group 2 was 6.0 (range: 1-22) (p<0.001). Tumor sizes range statistically significantly from 1 to 9.1 cm, with varying median values. The median for Group 2 was 3.0, while the median for Group 1 was 2.6 (p = 0.017), according to the results. Between 1 and 79 pelvic lymph nodes were excised; Group 2 had more nodes removed than Group 1. The average for Group 2 was 26.5, whereas the average for Group 1 was 15.8 (p<0.001), according to the data.

Between 1 and 48 para-aortic lymph nodes were excised in each group; there was no statistically significant variation in this number. With a p-value of 0.933, Group 1 had a median of 11 (range: 1-27), whereas Group 2 had a median of 11.5 (range: 1-48).

The lymph nodes that were removed ranged from 1 to 127. Furthermore, compared to Group 1, Group 2 had more lymph nodes removed overall. There was a statistically significant difference (p<0.001) between the means of Group 1 and Group 2. Group 1's mean was 23.3 (range: 4-44), whereas Group 2's mean was 37.3 (range: 1-127) (Table 3).

**Table 3:** Case distribution according to length of surgery, length of stay in the hospital after surgery, tumor diameter, number of pelvic lymph nodes, number of para-aortic lymph nodes, and overall number of lymph nodes. ^1^Independent two-sample t-test, ^2^Mann-Whitney U test

	Group 1 (n: 69)	Group 2 (n: 133)	p
	Mean ± SD	Mean ± SD	
Operation time (minutes)	241.3 ± 130.7	143.8 ± 69.8	<0.001^2^
Postoperative length of stay	4.0 ± 2.2	7.0 ± 4.3	<0.001^2^
Tumor diameter	2.6 ± 1.3	3.3 ± 1.9	0.017^2^
Number of pelvic lymph nodes	15.8 ± 5.9	26.5 ± 16.1	<0.001^1^
Number of paraaortic lymph nodes	12.4 ± 6.2	13.9 ± 9.9	0.933^2^
Total number of lymph nodes	23.3 ± 9.9	37.3 ± 23.9	<0.001^1^

Upon analysing the material extracted from the study participants, it was found that 46.5% (94) of the patients had grade 1 status, 47.5% (96) had grade 2 status, and 5.9% (12) had grade 3 status. All cases included in the study had endometrioid histology, and the groups' histology grades did not differ significantly (p = 0.626).

Following surgical staging, 137 patients, or 67.8% of the total, were classified as being in Stage 1A, 50 patients as being in Stage 1B, and 15 patients, or 7.4%, as being in Stage 2. The distribution of patients by surgical stage among the laparoscopy and laparotomy groups did not show any statistically significant differences (p = 0.204), indicating that the surgical approach did not influence staging outcomes.

In 28.7% (58) of the patients, there was no myometrial invasion. The myometrium was impaired in half or less in 44.1% (89) of the cases. In 27.2% (55) of the patients, the myometrial invasion was more than half. However, surgical staging is determined by multiple factors beyond myometrial invasion alone, including lymphovascular invasion, tumor grade, and other pathological criteria. Therefore, while myometrial invasion greater than 50% is often associated with Stage 1B, it does not automatically classify all such cases as Stage 1B. Consequently, the total number of Stage 1A cases (n=137) does not directly correspond to the sum of cases with no or minimal myometrial invasion. Myometrial invasion did not significantly differ across the groups (p = 0.087).

In 10.9% (22) of the patients, there was lymphovascular invasion. In 180 instances, or 89.1%, there was no evidence of lymphovascular invasion. Regarding the incidence of lymphovascular invasion, there was no statistically significant difference between the groups (p = 0.097) (Table 4).

**Table 4:** Surgical stages of the cases, distribution according to tumor grade, myometrial invasion degree and presence of lymphovascular invasion. ^1^Chi-square test

	Group 1	Group 2	p^1^
Grade	n = 69	n = 133	
Grade 1	29 (42%)	65 (48.9%)	0.626
Grade 2	36 (52.2%)	60 (45.1%)	
Grade 3	4 (5.8%)	8 (6%)	
Stage	n = 69	n = 133	
1A	52 (75.4%)	85 (63.9%)	0.204
1B	12 (17.4%)	38 (28.6%)	
2	5 (7.2%)	10 (7.5%)	
Myometrial invasion	n = 69	n = 133	
0	26 (37.7%)	32 (24.1%)	0.087
<1/2	29 (42%)	60 (45.1%)	
>1/2	14 (20.3%)	41 (30.8%)	
Lymphovascular invasion	n = 69	n = 133	
Yes	11 (15.9%)	11 (8.3%)	0.097
No	58 (84.1%)	122 (91.7%)	

Compared to Group 1, Group 2 required more intraoperative blood transfusions. In Group 1, two patients needed an intraoperative blood transfusion, whereas in Group 2, 18 patients needed one (p = 0.016). The higher transfusion rate in Group 2 is likely related to the surgical approach, as laparotomy is associated with greater intraoperative blood loss compared to laparoscopy. While patient-related factors such as baseline hemoglobin levels may play a role, the primary reason for this difference appears to be intervention-related.

Between the groups, there was no statistically significant variation in the incidence of intraoperative complications. Six patients in Group 2 and four individuals in Group 1 experienced intraoperative problems (p = 0.738). One patient had bladder damage, one patient had ureter damage, and two patients had vascular damage in the laparoscopic group; in Group 2, there was one bladder, one ureter, four bowel, and four vascular damage cases. Regarding the kinds of problems that transpired, there was no statistically significant difference (p = 0.12). Two patients (2.9%) with ureteric and vascular injury received laparotomy in the laparoscopic group.

Group 2 had a significantly higher rate of postoperative complications than the other groups. In Group 1, two patients had postoperative complications, whereas in Group 2, 17 patients did (p = 0.022). One patient in Group 1 experienced a fever 24 hours later, and another experienced DVT (deep vein thrombosis). Thirty-nine patients in Group 2 developed wound site infections, three patients had DVT, and five patients had fever 24 hours later. Regarding postoperative problems, there was a notable distinction between the two groups. The types of problems that occurred did not significantly differ across the groups (p = 0.335) (Table 5).

**Table 5:** The prevalence and kind of intraoperative problems, the distribution of postoperative complications, and the distribution of blood transfusion requirements in cases. ^1^Chi-square test, ^2^Fisher's Exact test

	Group 1	Group 2	p^1^
Blood Transfusion			
Yes	2 (2.9)	18 (13.5)	0.016
No	67 (97.1)	115 (86.5)	
Presence of intraoperative complications			
Yes	4 (5.8)	6 (4.5)	0.738^2^
No	65 (94.2)	127 (95.5)	
Intraoperative complications			
Bladder	1 (25)	0 (0)	0.120
Ureter	1 (25)	0 (0)	
Intestinal	0 (0)	4 (66.7)	
Vessels	2 (50)	2 (33.3)	
Presence of postoperative complications			
Yes	2 (2.9)	17 (12.8)	0.022
No	67 (97.1)	116 (87.2)	
Postoperative complications			
Fever after 24 hours	1 (50)	5 (29.4)	0.335
Wound infection	0 (0)	9 (52.9)	
Deep Vein Thrombosis	1 (50)	3 (17.6)	

Three deaths (4.3%) occurred in Group 1 and 17 deaths (12.8%) in Group 2. All deaths were disease-related, primarily due to cancer progression and recurrence rather than surgical complications. Relapses occurred in three patients (4.3%) in Group 1 and 12 patients (9%) in Group 2. Among relapsed cases, local recurrence was observed in one patient in Group 1 and two patients in Group 2, while the remaining relapses involved distant metastases.

The 5-year overall survival (OS) rates were 94.2% in Group 1 and 93.1% in Group 2, with no statistically significant difference (p = 0.703). Similarly, the 5-year disease-free survival (DFS) rates were 94.0% in Group 1 and 93.0% in Group 2, showing no significant difference between the two groups (p = 0.647). These findings indicate that both surgical approaches offer comparable long-term oncological outcomes. These details are summarized in Table 6.

**Table 6:** Comparison of the overall survival and disease-free survival rates during a 5-year mortality period across different groups. *Log Rank (Mantel-Cox) test

	Overall survival		Disease-free survival	
	Survival percentage ± SD	p	Survival percentage ± SD	p
Operation Type				
Group 1	94.2% ± 3.5%	0.703	94.0% ± 3.6%	0.647
Group 2	93.1% ± 22%		93.0% ± 2.2%	

**Figure 1:**
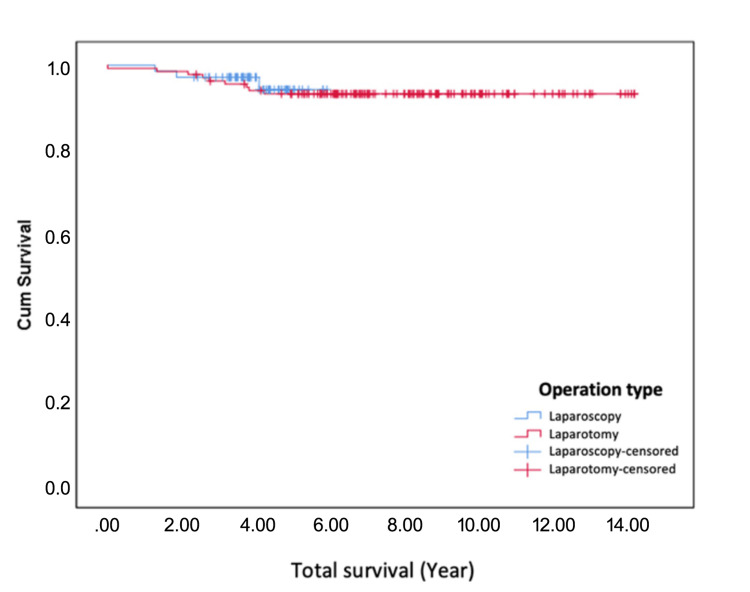
Kaplan-Meier curve showing overall survival (OS) rates for patients undergoing laparoscopy and laparotomy over a 5-year period. Censored data points are marked for both groups.

**Figure 2:**
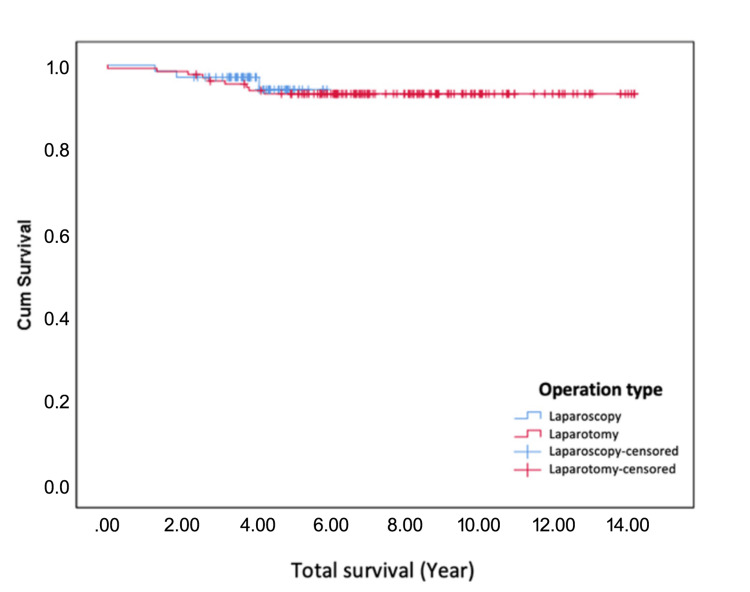
Kaplan-Meier curve showing disease-free survival (DFS) rates for patients undergoing laparoscopy and laparotomy over a 5-year period. Censored data points are marked for both groups.

In the Kaplan-Meier survival curves depicted in Figure 1 and Figure 2, the terms 'laparoscopic censored' and 'laparotomy censored' represent participants in the respective groups who were censored during the follow-up period. Censoring occurred when a participant did not experience the event of interest (e.g., recurrence or death) during the study, was lost to follow-up, or completed the study without experiencing the event. These censored data points are shown as specific symbols (e.g., crosses) on the survival curves, indicating the time at which the participant's data were no longer included in survival probability calculations.

## Discussion

The main method for managing and assessing endometrial cancer is surgery. The importance of surgical intervention has increased even more with the ability to detect and treat disease issues early. Laparoscopy's rising popularity in recent years has prompted research into its potential applications in oncological surgery. As a result, studies comparing the efficiency of laparoscopic and conventional laparotomy techniques have been conducted. Based on previously published research, this study sought to evaluate the reliability and effectiveness of several surgical approaches for the treatment of endometrioid endometrial cancer in its early stages.

In 2009, the Gynecologic Oncology Group (GOG) reported the results of the largest prospective randomised study on endometrial cancer, known as the LAP2 Phase III trial. A total of 1696 patients who underwent laparoscopy and 920 patients who underwent laparotomy participated in this study. The median operation duration was reported as 204 minutes for laparoscopy and 130 minutes for laparotomy [8]. The duration of the operation was found to be longer in the laparoscopy group. The average duration was calculated as 240 minutes in the laparoscopy group and 135 minutes in the laparotomy group. Our surgical durations were similar to those of other studies.

In a comprehensive meta-analysis published by Zhang et al. (2012), laparoscopy and laparotomy methods were compared in the treatment of endometrial cancer. In this meta-analysis, a total of 3599 patients from eight studies were included. The results of the study indicate that laparoscopy resulted in less intraoperative blood loss compared to other methods [9]. In the study, we discovered that the group that underwent laparoscopy required much fewer intraoperative blood transfusions.

Tozzi et al. conducted a randomised study on a group of 122 patients with endometrial cancer, in which 63 patients underwent laparoscopy and 59 patients underwent laparotomy [10]. The rate of postoperative complications in patients who underwent laparoscopy was found to be lower. In the study, laparoscopy had much fewer postoperative complications [11, 12].

In a long-term comparative study conducted by Zullo et al., the average length of hospital stay was found to be 3.0 days for the laparoscopy group and 6.9 days for the laparotomy group. It was noted that the hospital stay was shorter in the laparoscopy group [13, 14]. In the study, the postoperative hospital stay for patients was four days in the laparoscopy group and six days in the laparotomy group. We found the short duration of the postoperative hospital stay to be significant in the group that underwent laparoscopy.

Ruan et al., in their study consisting of 145 patients with early-stage endometrial cancer who underwent laparoscopy and 229 laparotomies, reported that the number of LNs removed was higher in the laparotomy group [15]. In our study, there was no difference in the number of para-aortic lymph nodes removed in both groups. The number of pelvic LN and total LN was higher in the laparotomy group.

The most significant issue in oncology surgery is intraoperative complications. One potential consideration in interpreting our findings is the impact of tumor grade on surgical and oncological outcomes. While our study included patients across all three histological grades of endometrioid adenocarcinoma, a subgroup analysis based on grade-specific survival and recurrence patterns could provide additional insights. Future research with larger sample sizes stratified by histological grade could offer a more detailed perspective on whether surgical outcomes vary significantly between low- and high-grade tumors. Walker et al. reported that the complication rate in laparotomy and laparoscopy was 8% in their study [8]. In our study, there was no difference in the rate of intraoperative complications between the two groups. Our rates were consistent with those in the literature.

The LACE study is a substantial, international randomised trial that compares 760 individuals diagnosed with stage I endometrial cancer who underwent either laparoscopy or laparotomy [16]. This study shows that there was no statistically significant disparity in disease-free survival or overall survival rates between cases where laparoscopy and laparotomy were conducted. Additionally, patients who underwent laparoscopy experienced a superior quality of life throughout the postoperative period [7, 17]. In their meta-analysis study, Galaal et al. [4] reported the results of nine studies evaluating a total of 4389 women. The study indicated that there is no difference in survival or disease-free survival between patients with early-stage endometrial cancer who undergo laparotomies [18, 19]. In our study, there was no difference in the 5-year overall survival and disease-free survival rates across the groups. Although recurrence rates were comparable between the groups, further analysis of recurrence patterns reveals potential differences in recurrence type. Although recurrence rates were comparable between the groups, different recurrence patterns may be influenced by tumor characteristics. Previous studies suggest that factors such as histological grade and lymphovascular invasion play a critical role in determining whether a recurrence presents as local or distant. While our study was not specifically designed to analyze these factors in detail, understanding their impact on recurrence patterns could provide valuable insights for future research. Larger, multicentric studies incorporating stratified analyses of these variables could help clarify their role in oncological outcomes. While our study was not designed to detect statistically significant differences in recurrence patterns, future research could explore whether histological grade, lymphovascular invasion, or other pathological factors influence the mode of recurrence.

Beyond oncological characteristics, patient-specific metabolic and hormonal factors, such as BMI, diabetes, and menopausal status, may influence surgical decision-making and perioperative outcomes. High BMI has been associated with increased technical difficulties in laparoscopic procedures, while diabetes and postmenopausal status could impact wound healing, infection risk, and overall recovery. Although our study did not perform a subgroup analysis based on these variables, future research should explore their role in determining the most suitable surgical approach for different patient profiles.

While our findings support previous literature showing comparable oncological outcomes between laparoscopic and open surgery, further analysis of our data highlights some important aspects of surgical intervention. The shorter hospital stay and reduced postoperative complications observed in the laparoscopic group suggest that minimally invasive surgery provides significant perioperative advantages without compromising oncological safety. Additionally, despite the longer operative time in laparoscopy, the reduction in intraoperative blood transfusion rates demonstrates an overall benefit in perioperative management. These findings support the continued shift towards minimally invasive approaches as the standard of care in appropriately selected cases.

Although our findings align with previous studies demonstrating the advantages of minimally invasive surgery, this study reinforces these conclusions with contemporary data from a well-defined patient population. A comprehensive review of the literature did not reveal any major studies reporting contradictory findings regarding the perioperative and oncological outcomes of laparoscopic versus open surgery in early-stage endometrial cancer. However, variations in study populations, surgical techniques, and follow-up protocols across different institutions may contribute to subtle differences in reported outcomes. Future large-scale, multicentric studies could help refine our understanding of whether certain patient subgroups may exhibit differing responses to surgical approaches. By providing updated evidence on laparoscopic and laparotomic approaches in early-stage endometrial cancer, our study contributes to the growing body of literature that supports the safety and efficacy of minimally invasive techniques. Future studies with larger multicentric datasets may further clarify the long-term oncological equivalence of these methods.

The most important limitation of the study is that it was conducted retrospectively. A retrospective design was chosen due to the availability of a well-documented institutional database covering an extensive period of patient follow-up. While prospective studies offer better control over variables, retrospective analyses allow the inclusion of a larger patient cohort with long-term outcomes, which enhances the robustness of our survival analysis.

However, the study has several strengths. All surgeries were performed by a single, experienced surgical team, ensuring consistency in surgical techniques and reducing variability in operator skill. Additionally, all pathological evaluations were conducted by the same team of gynecopathologists, which minimized interobserver variability in histopathological assessment. Additionally, the inclusion of only those cases operated on by surgeons with the same level of expertise minimizes bias related to operator-dependent variability. Moreover, an essential methodological strength of our study is that all gynecopathologists assessing the histopathological specimens were blinded to the surgical technique used. This ensured an unbiased evaluation of oncological parameters, further enhancing the reliability of our pathological assessments. These factors contribute to the reliability of our findings.

Despite these strengths, our study has some limitations. Being a retrospective, single-center study, the findings may have limited generalizability to broader populations. One important consideration in interpreting our findings is the potential influence of confounding factors. Although our study attempted to minimize bias by ensuring that all surgeries were performed by surgeons with equivalent expertise, individual patient characteristics such as age, BMI, comorbidities, and tumor biology may have influenced perioperative outcomes. Additionally, differences in postoperative care, including the administration of adjuvant therapies, could have impacted long-term survival and recurrence rates. While we employed rigorous data selection and analysis methods, future studies incorporating propensity score matching or multivariate regression analyses could further control for these variables and provide a more precise understanding of the factors influencing surgical outcomes. Selection bias may also be present due to the retrospective nature of patient allocation into surgical groups. Future prospective, multicentric studies with larger sample sizes and randomized allocation would help validate and expand upon our results.

However, the fact that all patients were performed by the same team experienced in laparoscopy and all materials were examined by the same gynecopathologists can be considered a strength of the study.

## Conclusions

This study compared laparoscopy and laparotomy in the management of early-stage endometrioid endometrial adenocarcinoma. Both approaches showed similar 5-year overall and disease-free survival rates, with no statistically significant differences. Laparoscopy, however, was associated with shorter postoperative hospital stays and fewer complications, highlighting its advantages as a minimally invasive option. These findings support the use of laparoscopy as a safe, effective, and patient-centered alternative to laparotomy in appropriately selected cases. With the continuous evolution of minimally invasive techniques, including robotic surgery and vaginal natural orifice transluminal endoscopic surgery (vNOTES), future research should focus on comparing these emerging approaches to further optimize surgical outcomes and patient safety.

## References

[1] Plaxe S, Mundt AJ (2021). Overview of resectable endometrial carcinoma. In: UpToDate.

[2] Zhao H, Xu Q (2020). Long non-coding RNA DLX6-AS1 mediates proliferation, invasion and apoptosis of endometrial cancer cells by recruiting p300/E2F1 in DLX6 promoter region. J Cell Mol Med.

[3] Ferlay J, Soerjomataram I, Dikshit R (2015). Cancer incidence and mortality worldwide: sources, methods and major patterns in GLOBOCAN 2012. Int J Cancer.

[4] Galaal K, Donkers H, Bryant A, Lopes AD (2018). Laparoscopy versus laparotomy for the management of early stage endometrial cancer. Cochrane Database Syst Rev.

[5] Fram KM (2002). Laparoscopically assisted vaginal hysterectomy versus abdominal hysterectomy in stage I endometrial cancer. Int J Gynecol Cancer.

[6] Scribner DR Jr, Walker JL, Johnson GA, McMeekin SD, Gold MA, Mannel RS (2001). Surgical management of early-stage endometrial cancer in the elderly: is laparoscopy feasible?. Gynecol Oncol.

[7] Chattopadhyay S, Galaal KA, Patel A, Fisher A, Nayar A, Cross P, Naik R (2012). Tumour-free distance from serosa is a better prognostic indicator than depth of invasion and percentage myometrial invasion in endometrioid endometrial cancer. BJOG.

[8] Walker JL, Piedmonte MR, Spirtos NM (2009). Laparoscopy compared with laparotomy for comprehensive surgical staging of uterine cancer: Gynecologic Oncology Group Study LAP2. J Clin Oncol.

[9] Zhang H, Cui J, Jia L, Hong S, Kong B, Li D (2012). Comparison of laparoscopy and laparotomy for endometrial cancer. Int J Gynaecol Obstet.

[10] Tozzi R, Malur S, Koehler C, Schneider A (2005). Laparoscopy versus laparotomy in endometrial cancer: first analysis of survival of a randomized prospective study. J Minim Invasive Gynecol.

[11] Gitas G, Pados G, Laganà AS, Guenther V, Ackermann J, Alkatout I (2023). Role of laparoscopic hysterectomy in cervical and endometrial cancer: a narrative review. Minim Invasive Ther Allied Technol.

[12] Asher R, Obermair A, Janda M, Gebski V (2018). Disease-free and survival outcomes for total laparoscopic hysterectomy compared with total abdominal hysterectomy in early-stage endometrial carcinoma: a meta-analysis. Int J Gynecol Cancer.

[13] Zullo F, Palomba S, Falbo A (2009). Laparoscopic surgery vs laparotomy for early stage endometrial cancer: long-term data of a randomized controlled trial. Am J Obstet Gynecol.

[14] Yin X, Shi M, Xu J, Guo Q, Wu H (2015). Perioperative and long-term outcomes of laparoscopy and laparotomy for endometrial carcinoma. Int J Clin Exp Med.

[15] Ruan XC, Wong WL, Yeong HQ, Lim YK (2018). Comparison of outcomes following laparoscopic and open hysterectomy with pelvic lymphadenectomy for early stage endometrial carcinoma. Singapore Med J.

[16] Obermair A, Janda M, Baker J (2012). Improved surgical safety after laparoscopic compared to open surgery for apparent early stage endometrial cancer: results from a randomised controlled trial. Eur J Cancer.

[17] Janda M, Gebski V, Brand A (2010). Quality of life after total laparoscopic hysterectomy versus total abdominal hysterectomy for stage I endometrial cancer (LACE): a randomised trial. Lancet Oncol.

[18] Chang-ji X, Jing Z, Peng G, Yang X (2014). Laparoscopy versus laparotomy for endometrial cancer: system review. Acta Metall Sin.

[19] Marcos-Sanmartín J, López Fernández JA, Sánchez-Payá J (2016). Does the type of surgical approach and the use of uterine manipulators influence the disease-free survival and recurrence rates in early-stage endometrial cancer?. Int J Gynecol Cancer.

